# Supplementary material for: A Short Series of Case Reports of COVID-19 in Immunocompromised Patients
Source: Viruses. 2022 Apr 29;14(5):934. doi: 10.3390/v14050934 (PMC9145915; doi:10.3390/v14050934)
Supplement: Supplementary file 1 [file viruses-14-00934-s001.zip › viruses-1601402-supplementary.pdf]

viruses

MDPI

Supplementary materials

A Short Series Case Reports of COVID-19 in Immunocompromised Patients

Table S1. Ct value, genome recovery (%), Variant and GISAID submission details of eight immunocompromised patients.

Patient ID	CII-Sample-ID	Sample Type	Collection Date (yyyy-mm-dd)	Ct values in qRT-PCR N1	Ct values in qRT-PCR N2	Ct values in qRT-PCR RP	% SARS-CoV-2 Genome recovery	GISAID Accession ID
Pt 1	SBA-001	serum	2021-04-06	31.07	29.93	25.82	99.75	EPI_ISL_4298277
	SBA-002	serum	2021-04-12	32.32	31.65	28.02	98.46	-
	SBA-003	serum	2021-04-19	Undetermined	Undetermined	27.48	-	-
Pt 2	SBA-010	serum	2021-04-14	Undetermined	Undetermined	32.40	-	-
	SBA-011	serum	2021-04-19	Undetermined	Undetermined	29.23	-	-
Pt 3	SBA-014	serum	2021-04-14	Undetermined	Undetermined	27.30	-	-
	SBA-015	serum	2021-04-18	Undetermined	Undetermined	27.79	-	-
Pt 4	SBA-016	serum	2021-04-14	Undetermined	Undetermined	29.27	-	-
	SBA-017	serum	2021-04-18	Undetermined	Undetermined	32.21	-	-
Pt 5	SBA-004	serum	2021-04-03	35.61	34.82	26.29	15.75	-
	SBA-005	serum	2021-04-08	Undetermined	Undetermined	25.26	-	-
Pt 6	SBA-006	serum	2021-04-03	Undetermined	Undetermined	28.68	-	-
	SBA-007	serum	2021-04-08	Undetermined	Undetermined	28.22	-	-
Pt 7	SBA-008	serum	2021-04-08	Undetermined	Undetermined	25.92	-	-
	SBA-009	serum	2021-04-13	Undetermined	Undetermined	26.41	-	-
Pt 8	SBA-012	serum	2021-04-14	Undetermined	Undetermined	28.17	-	-
	SBA-013	serum	2021-04-19	Undetermined	Undetermined	28.62	-	-
Pt 1	SBA-018	swab in VTM	2021-04-08	22.24	21.35	25.83	100.00	EPI_ISL_4298278
Pt 5	SBA-019	swab in VTM	2021-04-08	43.67	40.27	28.01	82.96	-
Pt 6	SBA-020	swab in VTM	2021-04-08	34.75	33.69	29.04	99.19	EPI_ISL_4298280
Pt 7	SBA-021	swab in VTM	2021-04-13	31.91	30.79	26.73	99.90	EPI_ISL_4298281
Pt 1	SBA-018-TC	Virus culture		22.00	21.80		100	EPI_ISL_4298279

Table S2. Immunological parameters (Erythrocyte sedimentation rate (ESR), C-Reactive Protein (CRP), White Blood Cell (WBC), Absolute Lymphocyte counts and Procalcitonin levels) during hospitalization of 8 immunocompromised patients.

	ESR (mm/hr) ref range <20		CRP (mg/dL) ref range <0.5		WBC (K/uL) (ref range 5-10)		Absolute Lymphocytes (ref range 0.8-5 K/uL)		Procalcitonin (ng/mL) ref range <0.15
Patient IDs	admission	discharge/death	admission	discharge/death	admission	discharge/death	admission	discharge/death	

Pt 1	85	17	23.4	0.8	18.7	8.7	240	90	6.27
Pt 2	32	15	<0.3	<0.3	2.18	4.35	110	220	0.12
Pt 3	50	11	26.4	0.3	43.5	37.6	5660	750	52.86
Pt 4	72	53	<0.3	<0.3	13.3	16.7	1180	540	0.24
Pt 5	25	33	13.2	1.2	2.79	28.8	310	270	0.62
Pt 6	25	62	6.5	12.1	11.1	17.2	440	690	0.12
Pt 7	114	48	28.4	3	13.2	15.8	930	430	0.44
Pt 8	41	13	16.7	0.7	9.39	6.84	420	930	0.14

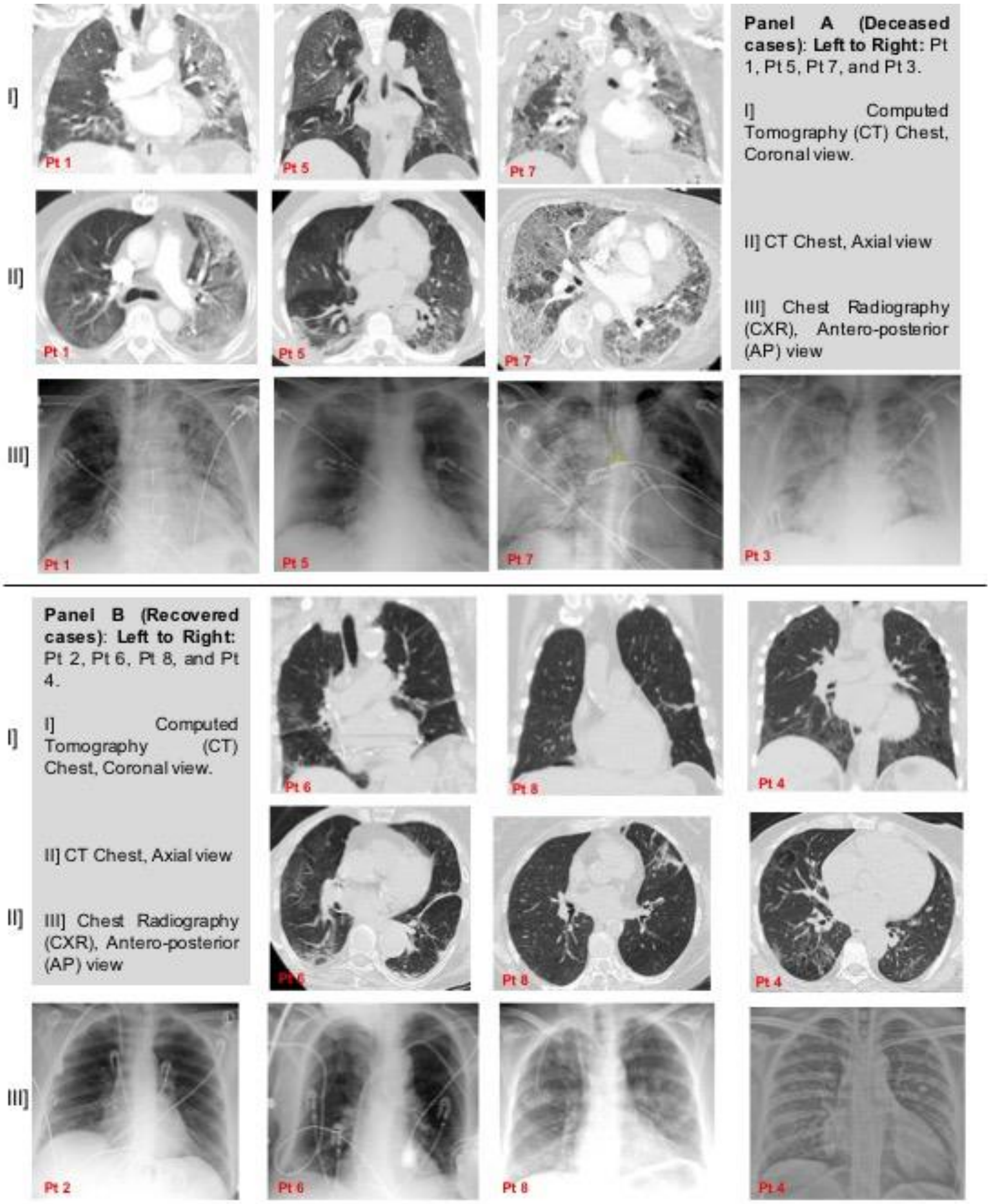

Figure S1. Computed tomography images (coronal and axial views) of deceased cases (**A**) and recovered cases (**B**) of 8 immunocompromised patients. Top panel A images show increased burden of diffuse multifocal ground glass opacities in bilateral lungs whereas bottom panel B images show mainly patchy airspace disease with intermittent areas of ground glass opacities.
